# Competing risks analysis for neutrophil to lymphocyte ratio as a predictor of diabetic retinopathy incidence in the Scottish population

**DOI:** 10.1186/s12916-023-02976-7

**Published:** 2023-08-10

**Authors:** Aravind Lathika Rajendrakumar, Simona M. Hapca, Anand Thakarakkattil Narayanan Nair, Yu Huang, Mehul Kumar Chourasia, Ryan Shun-Yuen Kwan, Charvi Nangia, Moneeza K. Siddiqui, Prathiba Vijayaraghavan, Shona Z. Matthew, Graham P. Leese, Viswanathan Mohan, Ewan R. Pearson, Alexander S. F. Doney, Colin N. A. Palmer

**Affiliations:** 1 Division of Population Health and Genomics, Ninewells Hospital, University of Dundee, Dundee, UK; 2 Biodemography of Aging Research Unit, Duke University, Durham, NC 27708‐0408 USA; 3 Division of Computing Science and Mathematics, University of Stirling, Stirling, FK9 4LA Scotland; 4 IQVIA, 3 Forbury Place, 23 Forbury Road, Reading, RG1 3JH UK; 5 Beatson Institute for Cancer Research, Glasgow, UK; 6 Wolfson Institute of Population Health, Queen Mary University of London, London, E1 4NS UK; 7 Madras Diabetes Research Foundation, Gopalapuram, Chennai India; 8 University of Edinburgh, Edinburgh, Scotland, UK; 9 Department of Medicine, Ninewells Hospital and Medical School, University of Dundee, Dundee, UK

**Keywords:** Diabetic retinopathy, Neutrophil–lymphocyte ratio, Competing risks, Subdistribution hazard ratio, Cause-specific hazard ratio

## Abstract

**Background:**

Diabetic retinopathy (DR) is a major sight-threatening microvascular complication in individuals with diabetes. Systemic inflammation combined with oxidative stress is thought to capture most of the complexities involved in the pathology of diabetic retinopathy. A high level of neutrophil–lymphocyte ratio (NLR) is an indicator of abnormal immune system activity. Current estimates of the association of NLR with diabetes and its complications are almost entirely derived from cross-sectional studies, suggesting that the nature of the reported association may be more diagnostic than prognostic. Therefore, in the present study, we examined the utility of NLR as a biomarker to predict the incidence of DR in the Scottish population.

**Methods:**

The incidence of DR was defined as the time to the first diagnosis of R1 or above grade in the Scottish retinopathy grading scheme from type 2 diabetes diagnosis. The effect of NLR and its interactions were explored using a competing risks survival model adjusting for other risk factors and accounting for deaths. The Fine and Gray subdistribution hazard model (FGR) was used to predict the effect of NLR on the incidence of DR.

**Results:**

We analysed data from 23,531 individuals with complete covariate information. At 10 years, 8416 (35.8%) had developed DR and 2989 (12.7%) were lost to competing events (death) without developing DR and 12,126 individuals did not have DR. The median (interquartile range) level of NLR was 2.04 (1.5 to 2.7). The optimal NLR cut-off value to predict retinopathy incidence was 3.04. After accounting for competing risks at 10 years, the cumulative incidence of DR and deaths without DR were 50.7% and 21.9%, respectively. NLR was associated with incident DR in both Cause-specific hazard (CSH = 1.63; 95% CI: 1.28–2.07) and FGR models the subdistribution hazard (sHR = 2.24; 95% CI: 1.70–2.94). Both age and HbA_1c_ were found to modulate the association between NLR and the risk of DR.

**Conclusions:**

The current study suggests that NLR has a promising potential to predict DR incidence in the Scottish population, especially in individuals less than 65 years and in those with well-controlled glycaemic status.

**Supplementary Information:**

The online version contains supplementary material available at 10.1186/s12916-023-02976-7.

## Background

Diabetic retinopathy (DR) is a major sight-threatening microvascular complication in individuals with diabetes [1]. It is estimated that close to 35% of diabetic patients continue to live with some form of diabetic retinopathy [2]. According to recent statistics, DR now contributes around 3.5% of all outcomes relating to severe vision loss and blindness in the United Kingdom [3]. On the other hand, a rising burden was noted globally in older age groups with more than 3.8 million experiencing vision problems from DR [4]. Despite knowledge regarding the clinical and genetic risk of DR, the heterogeneity in DR is yet to be fully uncovered [5]. For instance, the development and progression patterns for DR vary. Development and progression are sometimes independent of glycaemic control; glycated haemoglobin (HbA1c) or hypertension—two key risk factors for DR [6]. Systemic inflammation combined with oxidative stress are thought to capture most of the complexities involved in the pathology of diabetic retinopathy [7]. Indeed, epidemiological studies have found that increased activity of a pro-inflammatory enzyme, lipoprotein-associated phospholipase A_2_ (Lp-PLA2) was associated with both development and progression of DR [8]. However, the explicit role of the immune system in DR risk has not been explored in detail. Neutrophils and lymphocytes are white blood cells that play an important role in immunity [9]. The neutrophil–lymphocyte ratio (NLR) is a composite marker of inflammation which is routinely available as a part of clinical investigations [10]. NLR is widely used as a prognostic biomarker for many disease conditions such as predicting survival for multiple malignancies, diabetes, pneumonia, immune system dyscrasias and sepsis [11–16].

NLR is more robust to variations and provides more predictive information than its component markers [17, 18]. A high level of NLR is an indicator of abnormal immune system activity [19]. It represents subclinical inflammation—a prominent feature reported in chronic diseases and is generally high in individuals with diabetes [20, 21]. It was earlier proposed that the aetiology of diabetes is closely related to the activation of the innate immune system [22]. Diabetes mellitus (DM) downregulates the immune response by altering the structure and function of white blood components [23]. In diabetes, Neutrophils primarily act by secreting different inflammatory molecules that affect the integrity of blood vessels whereas lymphocytes act more as modulators of inflammatory activity [24–26].

Current estimates of the association of NLR with Diabetes and its complications are almost entirely derived from cross-sectional studies, suggesting that the nature of the reported association may be more diagnostic than prognostic [27, 28]. Deaths can be considered as a competing risk as it prevents the observation of DR in individuals with diabetes [8]. To our knowledge, no study has longitudinally examined the association between NLR and DR under a competing risks model. Also, temporal interaction effects of NLR and other diabetes risk factors such as HbA1c and age on the long-term incidence of DR have not been previously reported. Therefore, in the present study, we examined the utility of NLR as a biomarker to predict the incidence of DR in the Scottish population.

## Methods

### Study design

A retrospective analysis utilising the electronic medical records of Scottish patients living in Tayside and Fife with a clinical diagnosis of type 2 diabetes mellitus (T2DM) was conducted to evaluate the utility of NLR as a predictor of DR incidence. The DR records were electronically linked with T2DM diagnosis records previously curated and stored in the computing environment. The incidence of DR was defined as the time from baseline diagnosis of T2DM (T_0_) to the first diagnosis of R1 grade (background retinopathy) or greater in the Scottish retinopathy grading scheme [29]. Study duration was calculated as the time from T_0_ to the last follow-up visit or end of follow-up or death. The diabetic maculopathy status of the individuals was not considered for the analysis. The grades range from R0 to R4 which indicates increasing severity of DR from no retinopathy to severe proliferative DR. Prior to the analysis, it was decided that all records with laser photocoagulation would be marked as R4 and for incidence analysis, participants who already had retinopathy at baseline would be excluded. During analysis, both eyes were compared and the eye with the most advanced grade was considered for the analysis. To avoid other likely extraneous effects on NLR measurements, we also excluded all the individuals with NLR values above 20 at baseline from the analysis.

### Clinical covariates

The values of neutrophils and lymphocytes were extracted from the haematology file and NLR was calculated as the ratio between the absolute neutrophil count to the absolute lymphocyte count. The following clinical variables were included as covariates and extracted from haematology, demography, and biochemistry files through electronic linkage: age at diagnosis of T2DM, sex, glycated haemoglobin (HbA1c), body mass index (BMI), estimated glomerular filtration rate (eGFR), high-density lipoprotein cholesterol (HDL-c), and non–high-density lipoprotein cholesterol (non-HDL-c). No covariate values were imputed for this analysis. A diabetes drug covariate was also included defined as the use of tablets or insulin within 12 months from the diagnosis of T2DM (use of either tablet/insulin = 1 versus no use of diabetes drug = 0). All biochemical baseline parameters were summarised values (median) of the first 1–3 readings per participant (based on whichever is the highest available) obtained within 12 months before or after a diabetes diagnosis. NLR and other covariate readings closest to the baseline (within 12 months before or after diabetes diagnosis) were merged with the retinal screening data. We have excluded all NLR readings assayed after the diagnosis of malignancy or related treatment. Any NLR readings before the diagnosis of malignancies or chemotherapy and within the defined window period were included in the final summarised NLR value. For infectious diseases, any NLR readings following diagnosis within a window of one month (< = 31 days) were not considered, while the rest of the readings if present were included in the analysis. The latter was done to remove the possible bias in the readings arising due to infections. If there were multiple NLR readings close to 28 days, then only the first reading was included in the analysis. The information regarding the diseases and hospital visits was obtained by linking NLR data to the Scottish Morbidity Record of hospital admissions (SMR01).

### Statistical analysis

Competing risks are events that mask the observation of the main event of interest [30]. For instance, in the case of DR, an individual with diabetes and high levels of risk factors such as HbA1c will have an increased risk of developing DR [31]. However, events such as death remove the individual from developing DR [32]. This violates the normal assumption underlying usual survival analysis and necessitates consideration of deaths in the regression model. Two popular approaches, cause-specific hazard (CSH) and FGR are used to model the effect of covariates on the time-based outcome [33]. Another reason for preferring FGR over CSH is that the latter treats competing for events as censored and is less useful for interpreting survival probability [34]. Contrary to the CSH estimation, the subdistribution hazard (sHR) does not compute the estimate by removing the individuals experiencing the competing events from the risk set. Rather, the individuals with the competing event remain in the risk set and weights are assigned that take the contribution of their event time and censoring distribution into consideration [35]. However, under certain conditions, the CSH and corresponding Kaplan–Meier provide inflated probabilities especially if the frequency of competing events is high. In such scenarios, analysis based on cumulative incidence is shown to outperform the former [36].

In univariate analysis, continuous variables were expressed as mean (± SD) and as frequencies (percentages) for discrete variables; no imputations were performed. A *P* value < 0.05 was considered significant. For assessing the relative effect for NLR in DR incidence, the optimal cut-point was determined using a maximally selected rank statistics which returns the threshold that is best associated with the outcome [37]. Furthermore, NLR was used as a categorical variable in both FGR and CSH in backward stepwise competing risk models with the prior mentioned cut-off. CSH represent the relative effect of the risk factors on the hazards in event-free subjects and the FGR is more suitable for risk prediction as the covariate effect is modelled directly on incidence after accounting for competing events [38].

Follow-up time was limited to 10 years for DR events and deaths in the models considering the likely reducing ability of the marker to predict DR over a long follow-up period. Interactions were specified using a product term for NLR (categorical variable—high vs low) with age, HbA1C and eGFR. The risk estimates for the multivariate models pertaining to eGFR, SBP and DBP were transformed to reflect hazards per 10 mmHg. Similarly, the estimates for age and BMI were presented for 5 units. The interaction terms for the respective variables with NLR were computed with the same units mentioned above. The estimated probability for DR from the FGR was used to demonstrate the joint variation in risk associated with changing levels of NLR for different quartiles of HbA1c. We also conducted a stratified analysis to investigate NLR effects on DR across different age groups in individuals based on their glycaemic status. All statistical analyses were performed with R (version 3.5.1) software and associated packages [39–41].

## Results

### Selection of study participants

The data curation and final cohort selection process are represented in @Additional file 1: Fig. 1. Overall, screening information was available for 64,879 individuals in Tayside and Fife with diabetes of which 17.4% had some form of retinopathy at the first eye examination or at baseline. Among these, a total of 55,327 individuals had both diabetes diagnosis dates and censoring time information—which is the date they left the data catchment area, death, event date or end of the follow-up period (Additional file 1: Fig. 2). The final sample consisted of the records of 23,531 individuals with T2DM with complete covariate information which were available for incidence analysis with NLR (Additional file 1: Fig. 3).

### Baseline characteristics of the study population

The median follow-up time was 3.3 years. During the follow-up period of 10 years, 8416 had developed DR (35.8%) and 2989 (12.7%) were lost to competing events (death) without DR and 12,126 individuals did not have DR. In total, 11,405 out of 23,531 which is 48.4% of the sample experienced the event or competing event during the entire follow-up period. At the time of the first DR diagnosis, the majority of the DR cases, 8078 (95.9%) had R1 grade. The corresponding figures for the other advanced DR grades, R2, R3 and R4 were 192 (2.2%), 48 (0.5%) and 98 (1.1%), respectively. The demographic and clinical characteristics of the participants are provided in Table 1.

**Table 1:** Demographic and clinical characteristics of participants at baseline (*n* = 23,531)

Parameter	Mean	SD	Range
Age (years)	61.7	12.7	17.1–96.5
Male (%)	55.3		
Diabetes drug (yes %)	45.7		
HbA1c (%)	7.3	1.5	3.7–16.4
SBP (mmHg)	140.3	17.3	72–240
DBP (mmHg)	81.3	9.9	40–142.5
eGFR (ml/min/1.73m^2^)	80.6	19.6	15.5–163.2
Lymphocytes (10^9^/L)	2.3	1.62	0.3–187.2
Neutrophils (10^9^/L)	4.8	1.9	0.4–28.5
NLR	2.4	1.5	0.08–20.0
BMI (kg/m^2^)	32.2	6.6	15.2–73.9
HDL-c (mmol/L**)**	1.2	0.3	0.1–3.9
non-HDL-c (mmol/L)	3.7	1.1	0.6–18.4

Data are presented as mean ± SD or percentage (%); variables measured at the time of type 2 DM diagnosis

**Fig. 1:**
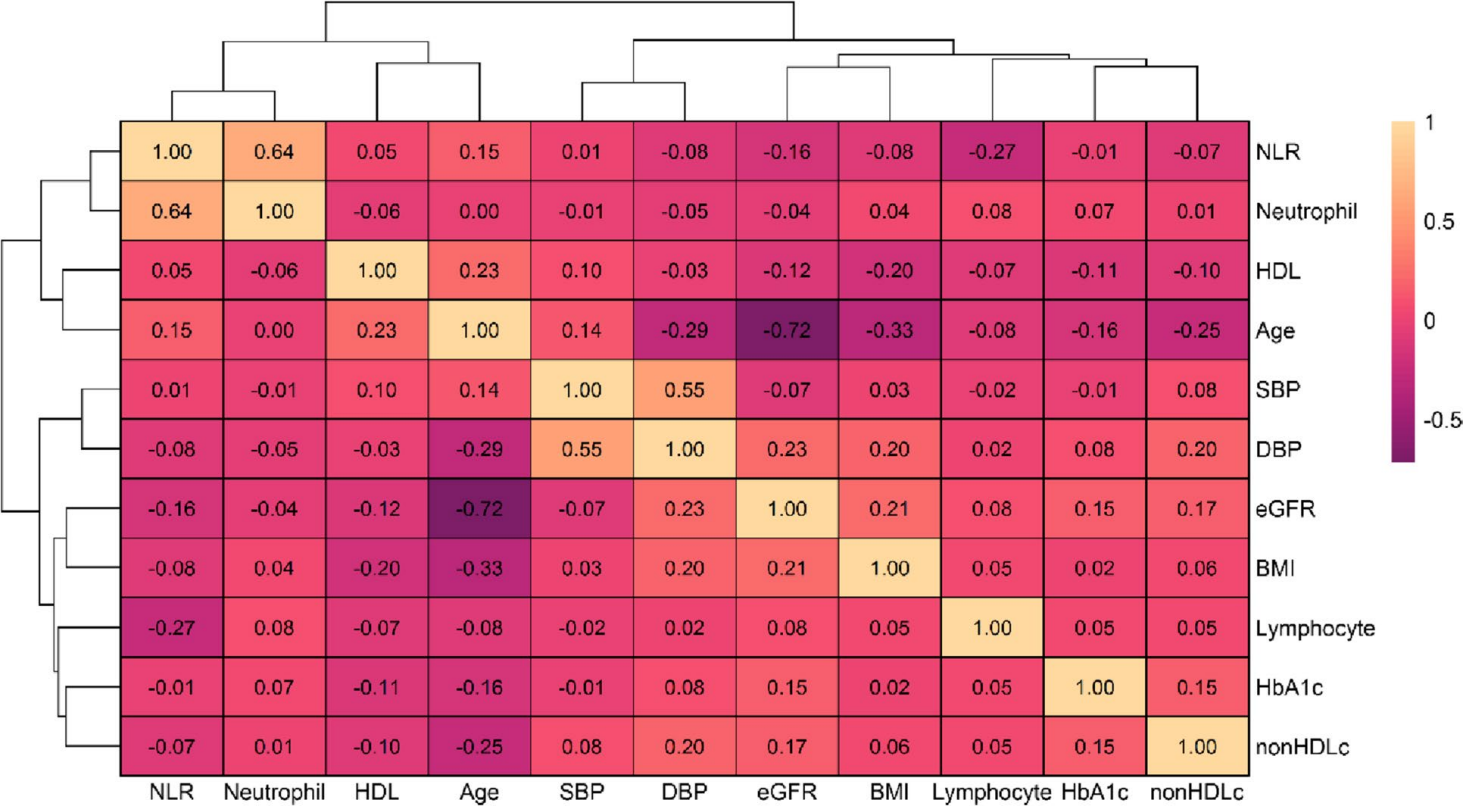
Heat map of Pearson correlation of NLR with demographic and clinical covariates at baseline

The participants were predominantly males (55.3%) and the mean (SD) age was 61.7 (12.7) years. Among these, 10,766 (45.7%) were prescribed diabetes drugs at baseline out of which 380 (3.5%) were on insulin and 8489 (78.8%) were on metformin. The median (IQR) level of NLR was 2.04 (1.5 to 2.7) and corresponding values for neutrophils and lymphocytes were 4.5 (3.5 to 5.6) 10^9/L and 2.2 (1.7 to 2.7) 10^9/L, respectively. Median NLR levels were high at the time of diagnosis of diabetes in individuals who went on to develop DR 2.0 (1.5 to 2.7) and in patients who died without DR 2.4 (1.7 to 3.4) relative to individuals who remained alive and did not develop DR 1.94 (1.5 to 2.5). The mean difference was statistically significant between the groups (*p* < 0.01). The Pearson correlation of NLR with other clinical covariates at baseline is provided in Fig. 1.

After accounting for competing risks at 10 years, the cumulative incidence of DR and deaths without DR derived from FGR analysis was 50.7% and 21.9%, respectively.

### Estimates from the competing risk models of incident DR

**Fig. 2:**
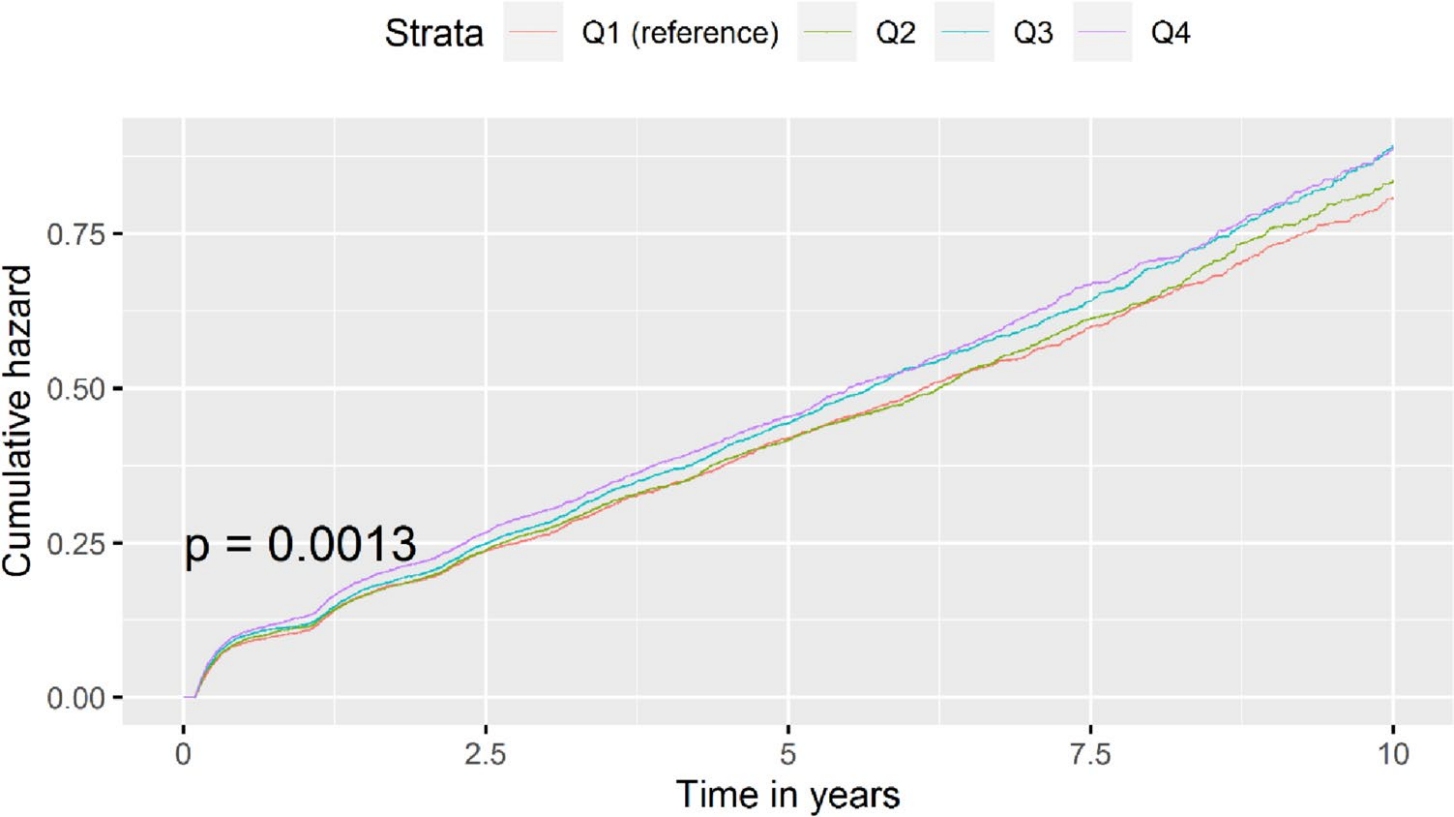
Cumulative incidence plot showing 10-year DR incidence risk for NLR quartiles in Tayside

The quartile-wise risk associated with increased NLR for DR was tested using the CSH model. Q1 denotes the first quartile and Q4 denotes the uppermost quartile. The cumulative incidence plot in Fig. 2 shows an increasing hazard for the incidence of DR with Q3 and Q4 conferring substantially higher risk than those in Q1.

**Fig. 3:**
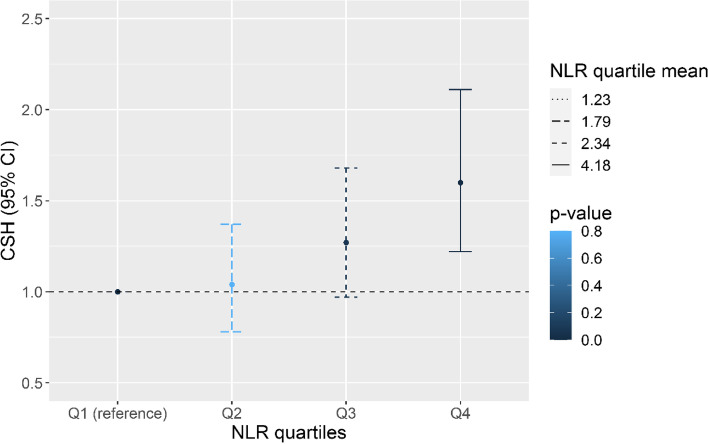
Adjusted Hazards for incidence of DR for different quartiles of NLR from the CSH Model. The model was adjusted for age, sex, HbA1c, eGFR, BMI, HDL, non-HDL-c, SBP, DBP, diabetes drug use at baseline, interaction terms for NLR quartiles with age, eGFR and HbA1c. Q1 denotes the first quartile and Q4 denotes the uppermost quartile. Quartile means for Q1–Q4 were 1.23, 1.79, 2.34 and 4.18, respectively. The Q1 is the reference category

The crude effects were further adjusted for all covariates and interaction terms for the NLR quartile with age, HbA1c and eGFR. Among the interaction terms, only the interaction term for NLR quartiles with HbA1c remained significant in the final model. A clear dose–response relationship was visible for the hazards for DR incidence for each quartile increase in NLR. The CSH for individuals in the higher NLR quartile was 1.60 (95% CI: 1.21–2.11, *p* < 0.001) relative to the referent lowest quartile (Fig. 3). Among the interaction terms for NLR quartiles with age, eGFR and HbA1c, only the latter retained significance in the model.

An optimal binary NLR cut-off value to predict retinopathy incidence was determined by maximal rank statistics, and it was 3.04. NLR above 3.04 was coded as high and risks were estimated based on this cut-off. Event-free survival (EFS) and cumulative incidence function (CIF) plots for NLR and other covariates are shown in Additional file 1: Figs. 4–5. Overall, 4406 (18.7%) individuals had high (> 3.04) NLR values. The estimates from the multivariate analysis for the CSH and FGR models for the 10-year incidence of DR are given in Tables 2 and 3.

**Table 2:** Results of the CSH regression model for 10-year incidence of DR (*n* = 23,531)

Parameter	Crude CSH (95% CI)	*P*	Adjusted CSH (95% CI)	*P*
Sex (M)	1.07 (1.02, 1.12)	< 0.01^**^	1.08 (1.03, 1.13)	< 0.001^***^
DBP (mmHg)	0.97 (0.94, 1.0)	0.07	0.97 (0.94, 0.99)	< 0.05^*^
HbA1c (%)	1.15 (1.11, 1.20)	< 0.001^***^	1.15 (1.11, 1.20)	< 0.001^***^
HbA1c × NLR^#^	0.94 (0.91, 0.97)	< 0.001^***^	0.94 (0.91, 0.97)	< 0.001^***^
SBP (mmHg)	1.05 (1.03, 1.06)	< 0.001^***^	1.05 (1.03, 1.06)	< 0.001^***^
Age × NLR^#^	0.99 (0.99, 1.0)	0.30	0.99 (0.99, 0.99)	< 0.001^***^
NLR (> 3.04)	2.32 (1.21, 4.43)	< 0.05^*^	1.63 (1.28, 2.07)	< 0.001^***^
non-HDL-c (mmol/L)	0.82 (0.75, 0.91)	< 0.001^***^	0.82 (0.75, 0.91)	< 0.001^***^
BMI (kg/m^2^)	0.95 (0.94, 0.97)	< 0.001^***^	0.95 (0.94, 0.97)	< 0.001^***^
eGFR (ml/min/1.73 m^2^)	1.03 (0.98, 1.08)	0.19	-	-
Diabetes drug (yes)	1.11 (1.05, 1.17)	< 0.001	1.11 (1.06, 1.17)	< 0.001^***^
Age (years)	1.02 (0.98, 1.06)	0.17	-	-
eGFR × NLR^#^	0.98 (0.94, 1.01)	0.26	-	-

Variables labelled with # represent variable interaction terms with NLR (product term represented x symbol between variables). The effect estimates for eGFR, SBP and DBP are presented for a 10-unit increase whereas age and BMI were shown for a 5-unit increase. The same applies to the interaction terms of these variables with NLR in the multivariate regression models

*CSH*, Cause-specific hazard ratio

^*^p < 0.05; ^**^p < 0.01; ^***^p < 0.001

**Table 3:** Results of the FGR model for 10-year incidence of DR (*n* = 23,531)

Parameter	Crude sHR (95% CI)	*P*	Adjusted sHR (95% CI)	*P*
Sex (M)	1.07 (1.02, 1.12)	< 0.01**	1.05 (1.01, 1.10)	< 0.05^*^
DBP (mm Hg)	0.98 (0.95, 1.01)	0.22	-	-
HbA1c (%)	1.19 (1.15, 1.24)	< 0.001^***^	1.19 (1.15, 1.24)	< 0.001^***^
HbA1c × NLR^#^	0.93 (0.90, 0.96)	< 0.001^***^	0.94 (0.90, 0.96)	< 0.001^***^
SBP (mmHg)	1.00 (1.00, 1.01)	< 0.001^***^	1.08 (1.06, 1.09)	< 0.001^***^
Age × NLR^#^	0.97 (0.94, 1.00)	0.05	0.98 (0.97, 0.98)	< 0.001^***^
NLR (> 3.04)	2.41 (1.27, 4.57)	< 0.01	2.24 (1.70, 2.94)	< 0.001^***^
non-HDL-c (mmol/L)	1.04 (0.95, 1.15)	0.35	-	-
BMI (kg/m^2^)	0.99 (0.99, 0.99)	< 0.001^***^	0.93 (0.91, 0.94)	< 0.001^***^
eGFR (ml/min/1.73 m^2^)	0.96 (0.92, 1.01)	0.16	0.97 (0.95, 0.98)	< 0.001^***^
Diabetes drug (yes)	1.01 (0.97, 1.07)	0.45	-	-
Age (years)	1.01 (0.97, 1.04)	0.60	-	-
eGFR × NLR^#^	1.00 (0.97, 1.04)	0.80	-	-

Variables labelled with # represent variable interaction terms with NLR. The effect estimates for eGFR, SBP and DBP are presented for a 10-unit increase whereas age and BMI were shown for a 5-unit increase. The same applies to the interaction terms of these variables with NLR in the multivariate regression models (product term represented by × symbol between variables)

*sHR*, sub hazard ratio

^*^p < 0.05; ^**^p < 0.01; ^***^p < 0.001

**Fig. 4:**
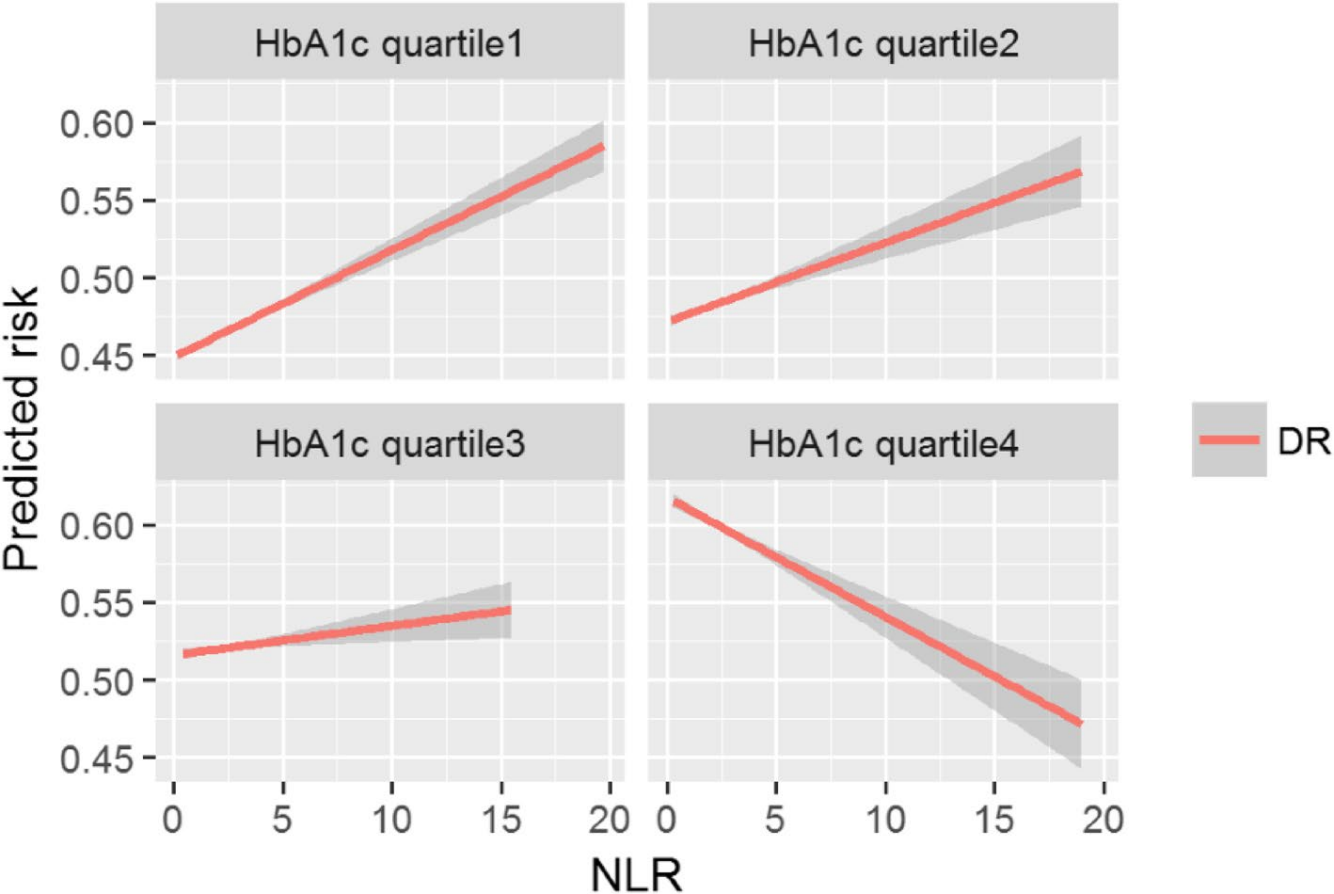
Risk for DR predicted by NLR stratified by quartiles of HbA1c estimated from covariate-adjusted FGR model (Hba1c quartile 4 had the highest HbA1c). The predicted risk was computed for each individual using the adjusted FGR model and the estimates were plotted for corresponding HbA1c quartile and NLR values. Note. The red line represents the model predicted risk for diabetic retinopathy for each unit increase in NLR stratified by quartiles of HbA1c. The slope of the red line shows the predicted risk. The width of the grey line represents the 95% confidence interval limits

Both models confirmed the increased risk for DR incidence in individuals with higher NLR values. The estimated hazard ratio for the CSH model was 1.63 (95% CI: 1.28, 2.07, *p* < 0.001) and sHR 2.24 (95% CI 1.70, 2.94, *p* < 0.001) for the FGR respectively. Notably, an interaction term for HbA1c and age with NLR was significant in both the models. Thus, there is a clear evidence of effect modification by HbA1c and age on the risk of DR predicted by NLR. Due to this, for better understanding, we provided more information to report the DR risk predicted by NLR across the strata of HbA1c and age rather than simply providing an average CSH or sHR point estimate (Fig. 4 and Table 4). To elaborate on the findings, we found that the individuals having high NLR levels and who were within the first or second quartiles of HbA1c had an increased risk of DR compared with those in quartile 3 or quartile 4. Figure 4 shows that the DR risk begins to reverse in quartile 3 and there is a total reversal of effect in quartile 4 where individuals having lower values of NLR levels were more likely to develop DR. We further investigated whether the DR risk predicted by NLR across 3 age groups (< = 45 years, > 45 and < 65 years, >  = 65 years) would differ in those with better glycaemic control (< = 7.0%). The highest risk was noted for <  = 45 years with this subgroup having a 45% higher failure rate than in individuals with NLR values below 3.04. An equivalent analysis for >  = 65 years, however, showed that the sHR attenuated to 1.10 (95% CI: 0.99, 1.22, *p* = 0.05) and was not statistically significant. Therefore, NLR may not be useful as a predictive biomarker in individuals having age above 65 years with better glycaemic control.

**Table 4:** Subgroup analysis of NLR showing the difference in predicted risk for DR in relatively younger (< = 45 years), (> 45 and < 65 years) and versus older (> = 65 years) age groups with better glycaemic control (< = 7.0%)

Variable	Age (< = 45 years)	*P*	Age (> 45 and < 65 years)	*P*	Age (> = 65 years)	*P*
	(*n* = 1055)		(*n* = 5819)		(*n* = 6285)	
NLR (> 3.04)	1.45 (1.08, 1.94)	< 0.05^*^	1.19 (1.05, 1.35)	< 0.01^**^	1.10 (0.99, 1.22)	0.05

Adjusted for sex, SBP (mmHg), DBP (mm Hg), BMI (kg/m^2^), non-HDL-c(mmol/L), HDL-c(mmol/L**)**, diabetes drug, and eGFR (ml/min/1.73 m^2^)

^*^p < 0.05; **p < 0.01

## Discussion

In our study, we have shown a powerful relationship between NLR levels at diagnosis of diabetes and the risk of retinopathy over a 10-year period. As NLR is a predictor for overall vascular morbidity and mortality, and retinopathy can be considered as a “survivor” phenotype, we have used both standard and “competing risk” methodologies to confirm that this relationship is independent of overall effects on mortality. Both models provide essential information pertaining to causality and prognosis and hence were reported in this study [38, 42]. Careful analysis of the relationship of NLR with known major DR risk factors revealed that NLR was directly correlated with both age and HbA1c levels, but that NLR demonstrated an interaction with both of these important risk factors when considering the risk for DR. In fact, the relationship of NLR with DR in those at the highest quartile of HbA1c was strongly reversed, suggesting a potential heterogeneity in mechanisms of DR under conditions of poor glycaemic control. This reveals that increased NLR may not be an ideal predictor of DR incidence risk in individuals with extremely high HbA1c values and age above 65 years measured at the time of T2DM diagnosis.

DR continues to be a major health problem despite a reduction in the prevalence of advanced forms seen over the last several years in Tayside [29]. Retinopathy incidence in diabetes depends on the duration of diabetes with most subjects highly likely to develop the condition over a 20-year course [43]. Hence, following DR incidence across 10 years allows sufficient time for properly characterising the condition [44, 45]. Generally, the Scottish retinal grading scheme is more concise compared to the other DR grading schemes. Also, as with the other grading schema, the DR category is ascertained on a progressive scale based on the clinical features. The clinical signs of DR such as microaneurysms and blot haemorrhages are visible from the R1 stage itself. As such, one of the determining features that differentiate between R1 to R3 includes more regional spread in the number of blot haemorrhages. Hence, we believe that while R2 and above are more robust sub forms of DR, ignoring R1 grade in survival would be incorrect and lead to a biased survival time. Identifying a suitable biomarker for the prediction of DR will be of utility in determining high-risk individuals beyond glycaemic control. NLR is suggested to be a good proxy marker for inflammation and endothelial dysfunction, both of which are hallmarks of diabetic retinopathy [46, 47].

The hypothesis linking endothelial abnormalities to inflammatory activity in diabetic retinopathy was confirmed in the Hoorn study [7]. Diabetes contributes to inflammation by activating biochemical pathways that result in the increased production of several inflammatory proteins and leukostasis [48]. Also, increased production of white blood cells is shown to enhance cytokine secretion in the retina further accelerating the inflammation-related damage in the retina [49]. Moreover, increased blood sugar levels were previously correlated with poor immunity and the administration of insulin was associated with higher neutrophil activity in diabetes [50, 51].

Besides the effect of NLR, remarkably, increasing levels of non-HDL-c and BMI were protective of DR in our analysis. Our finding regarding the effect of BMI is in agreement with the previous meta-analysis by Zhou et al. [52]. Most previously reported associations between non-HDL-c and retinopathy were on macular oedema than DR [53]. Our findings are in line with results of the Chennai Urban Rural Epidemiology Study (CURES) Eye Study which indicate that lipids may have a minor role in DR incidence [54]. Strengths of our study include population-level screening data and availability of longitudinal measures for NLR and other clinical covariates. Few studies have previously performed a time-based analysis of the effects of NLR on DR incidence by modelling death as a competing event in a large number of individuals with diabetes. Importantly, this analysis shows that the association between NLR and DR is not necessarily linear. We also observed that NLR values were high in some participants even when they had lower HbA1c and vice versa. A higher NLR threshold of 4.778 was determined recently by a cross-sectional study of the National Health and Nutrition Examination Survey (NHANES) population, which also showed a plateau effect for DR risk above this number and had a lower effect size in comparison to our work [56]. Even though our study also found evidence for the plateau effect, it had many advantages over the published report since our analysis was much larger, longitudinal in nature, and importantly, took competing risks into account. In addition, the investigators did not perform the stratified analysis for NLR with HbA1c, the main risk factor for DR. The analysis did display a similar attenuation of the role of NLR in the older individuals, as we have observed here, however, this difference was not significant. Based on our survival data, we have suggested a lower NLR cut-off of 3.04; however, this has to be considered in the context of both age and HbA1c levels.

As expected, HbA1c increase was associated with an increased probability to develop DR in our study. However, we found a significant protective association for high values of NLR and HbA1c denoted by the interaction term in CSH and FGR models. The introduction of the interaction term did not affect the significance of the main effects of HbA1c. Additionally, the main effect of age was not significant in either CSH or FGR models. Therefore, we chose to present the effect modification across categories of age and HbA1c. We showed that a statistically significant heterogeneity exists in DR risk predicted by NLR for higher age and HbA1c subgroups. The respective estimates were also controlled for relevant confounders of DR. Our findings may have implications for personalised medicine when using NLR as a risk stratification biomarker for DR. We illustrate that the relation between DR and NLR cannot be fully investigated without checking the interaction with HbA1c and age. The main limitation of our study is that NLR is highly variable and is influenced by a variety of clinical conditions such as physiological stress along with the possible unmeasured confounders resulting from the observational nature of the data [55]. However, we have attempted to address some of these issues by taking a summary of multiple readings (median) that may be more ideal than single-point measure. Furthermore, possible conditions affecting NLR such as malignant conditions, infections and outlier values were excluded to optimise the effects of NLR on DR risk. To the best of our knowledge, such extensive data curation for NLR has not been previously undertaken for DR analysis. Future studies may be conducted to elaborate on the different pathways, fluctuations and dependencies relating to NLR and its regulation in diabetes. Another interesting analysis could be joint modelling of the association between DR and NLR longitudinal values. We recommend validating our findings in multiple ethnicities with a much larger sample size to have sufficient statistical power in order to detect biologically relevant interaction effects.

## Conclusions

In conclusion, NLR is associated with DR risk, but due to the strong relationship of HbA1c levels and age with both DR risk and NLR levels, NLR seems to only provide additional useful information for identifying individuals at high risk for DR among those with clinical parameters that would be associated with low risk of DR, i.e., NLR is a good predictor for DR incidence only in those with well-controlled glycaemic status and below 65 years of age. Further works, including Mendelian randomisation studies and replication studies, are required to generalise the results of our study. The role of inflammation in DR pathogenesis as elicited by NLR may be explored as a therapeutic option to treat DR in the future.

### Supplementary Information

**Additional file 1:** **Figure S1. **Study flow diagram. **Figure S2.** Overall Survival for the incidence of DR in Tayside and Fife. **Figure S3.** Survival for the incidence of DR in Tayside and Fife for the analysed cohort. **Figure S4.** Event-free survival (EFS) plots of NLR covariates for 10-year DR incidence in Tayside and Fife. **Figure S5.** Cumulative Incidence Function (CIF) Plot of NLR and covariates for 10-year DR incidence in Tayside and Fife.

**Additional file 2.** STROBE Checklist for observational studies.

## Data Availability

Tayside and Fife electronic health data is maintained by the University of Dundee Health Informatics Centre (HIC) and is available in the Scottish Government accredited secure safe haven. The data is not currently available in the public domain. Researchers need to obtain prior approval from the HIC for accessing the data.

## Abbreviations

BMI: Body mass index
CHD: Coronary heart disease
CI: Confidence interval
CIF: Cumulative incidence function
CURES: Chennai Urban Rural Epidemiology Study
DBP: Diastolic blood pressure
DR: Diabetic retinopathy
eGFR: Estimated glomerular filtration rate
HbA1c: Glycated haemoglobin
HDLc: High-density lipoprotein cholesterol
HR: Hazard ratio
LDL-C: Low-density lipoprotein -cholesterol
NHANES: National Health and Nutrition Examination Survey
NLR: Neutrophil–lymphocyte ratio
Non-HDL-c: Non-high-density lipoprotein cholesterol
SBP: Systolic blood pressure
SD: Standard deviation
sHR: Sub hazard ratio
