# Supplementary material for: Competing risks analysis for neutrophil to lymphocyte ratio as a predictor of diabetic retinopathy incidence in the Scottish population
Source: BMC Med. 2023 Aug 10;21:304. doi: 10.1186/s12916-023-02976-7 (PMC10413718; doi:10.1186/s12916-023-02976-7)

**SUPPLEMENTARY FIGURES**

**Neutrophil to Lymphocyte Ratio as a Predictor of Diabetic Retinopathy Incidence in the Scottish population**

Aravind Lathika Rajendrakumar, PHD,^1,2^, Simona M Hapca, PHD,^1, 3^_,_ Anand Thakarakkattil Narayanan Nair, PHD,^1^, Yu Huang, PHD_,_^1^ _,_ Mehul Kumar Chourasia, PHD,^1,4^_,_ Ryan Shun-Yuen Kwan, PHD,^1,5,^ Charvi Nangia, PHD,^1^_,_ Moneeza K Siddiqui, PHD,^1,6^_,_ Prathiba Vijayaraghavan, MD,^7^_,_ Shona Z Matthew, PHD,^8^_,_ Graham P Leese, MD,^9^_,_Viswanathan Mohan, MD,^7^_,_ Ewan R Pearson, PHD,^1^, Alexander S F Doney, MD,^1^, Colin N A Palmer^*^, PHD,^1^

1. *Division of Population Health and Genomics, Ninewells Hospital, University of Dundee, Dundee, UK*
2. *Biodemography of Aging Research Unit, Duke University, Durham, NC, 27708‐0408, USA*
3. *Division of Computing Science and Mathematics, University of Stirling, Stirling FK9 4LA*
4. *IQVIA, 3 Forbury place, 23 Forbury Road, Reading, RG1 3JH, UK*
5. *Beatson Institute for Cancer Research, Glasgow, UK*
6. *Wolfson Institute of Population Health, Queen Mary University of London, E1 4NS*
7. *Madras Diabetes Research Foundation, Gopalapuram, Chennai, India*
8. *University of Edinburgh, Edinburgh, Scotland, UK*
9. *Department of Medicine, Ninewells Hospital and Medical School, University of Dundee, UK*

Figure 1. Study Flow Diagram

Tayside and Fife Eye Screening Data

(50, 9814 Records, n= 67,926 individuals)

12,599 participants were excluded:

No recorded DR grade (54,452 records, 1,729 individuals), First screening visit >=1990-01-01 (18,922 Records, 1,318 individuals), No T2DM diagnosis date (9,151 individuals), Excluded participants with date mismatch (follow-up, DM diagnosis, deaths) = 401 individuals

No recorded DR grade (45,5records, 66,197 PROCHI)

First screening visit >=1990-01-01 (43,6440 PROCHI, 64,879 PROCHI)

No DM diagnosis date -55728

Date out < DM diagnosis date:55616

Death date > DM diagnosis date:55612

Follow-up date > T2D date (55,327 PROCHI)

Sample size after quality control

(n=55,327 individuals)

31,796 participants were excluded due to no baseline information or based on inclusion criterion:

Excluded participants with no NLR information: 27,691 individuals, Excluded participants with no HbA1c information: 844 individuals, Excluded participants with no BP information: 471 individuals, Excluded participants with no eGFR information: 29 individuals, Excluded participants with no Lipid information: 543 individuals, Excluded participants with no BMI and baseline diabetes drug information: 1,520 individuals, Excluded NLR<=20: 32 individuals, Excluded participants with DR diagnosis at baseline: 666 individuals

Analysed cohort (n=23,531 individuals)

Figure 2. Overall Survival for the incidence of DR in Tayside and Fife (n=55,327)


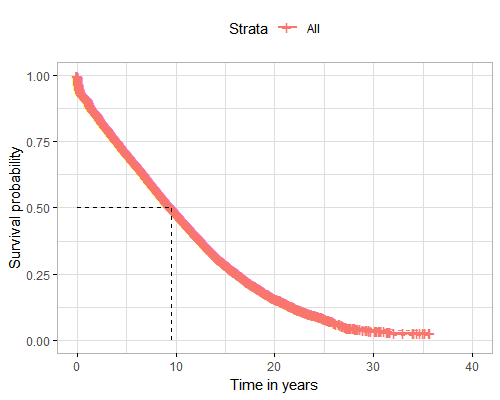


Incident DR =26,214, Median survival time = 9.55 years (95% CI: 9.42 years, 9.68 years)

Figure 3. Survival for the incidence of DR in Tayside and Fife for the analysed cohort (n=23,531)


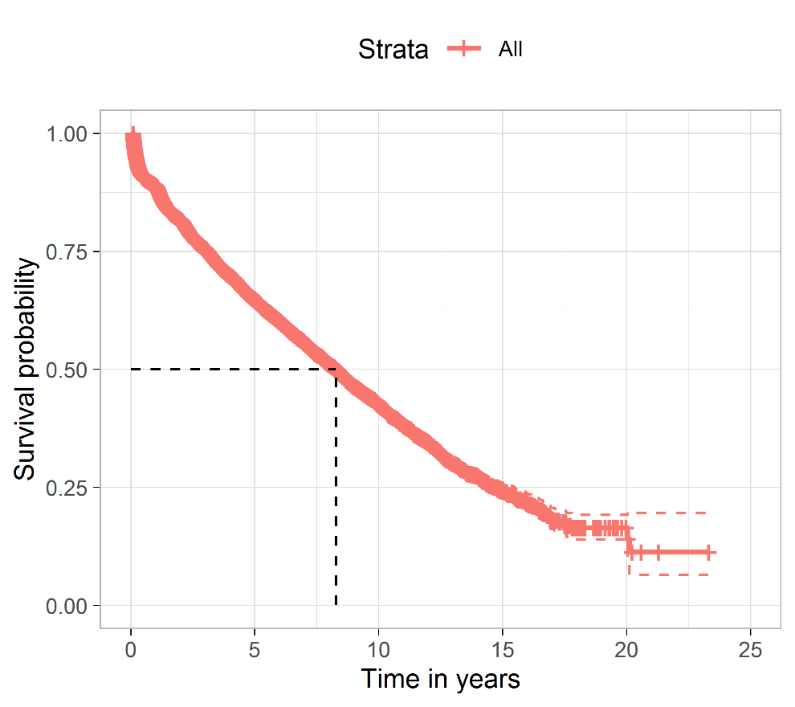


Incident DR =8,876, Median=8.27 years (95% CI: 8.08 years, 8.48 years years**)**

Figure 4. Event-free survival (EFS) plots showing the risk associated with demographic and clinical covariates for experiencing DR for a 10 year follow-up period in Tayside and Fife (n=23,531)


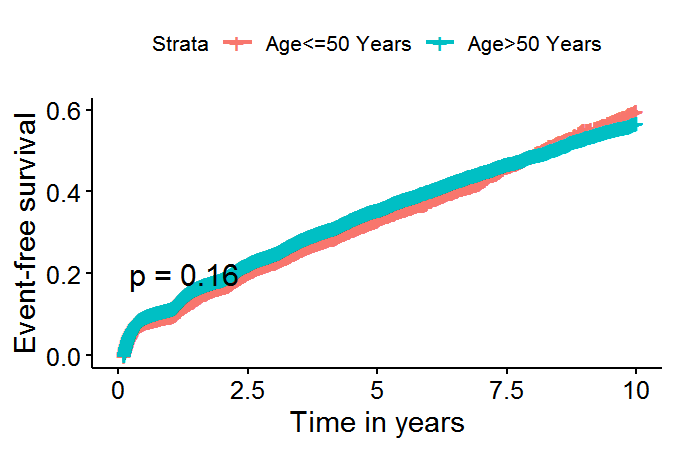

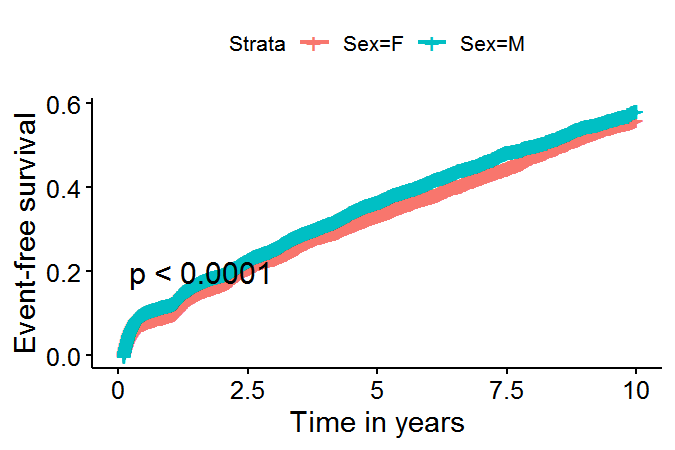


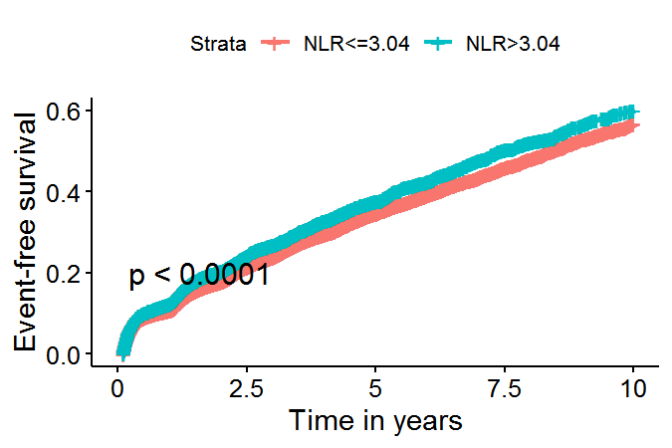

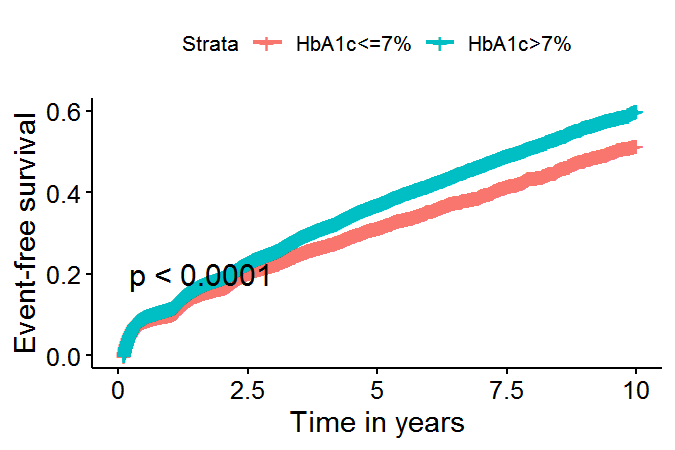


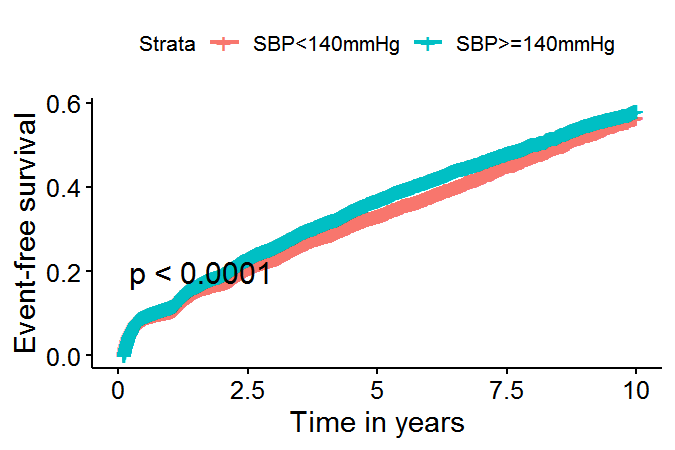

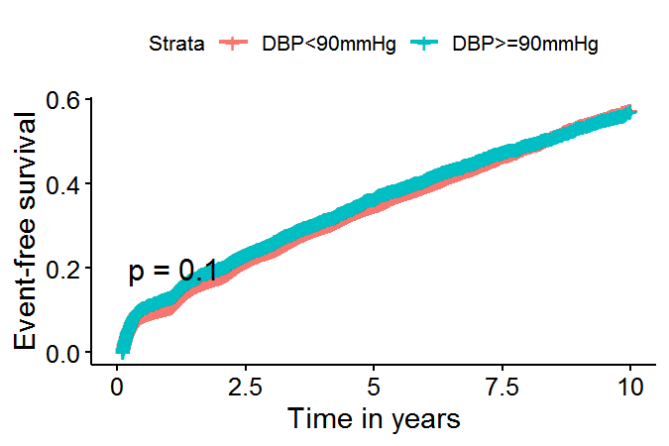


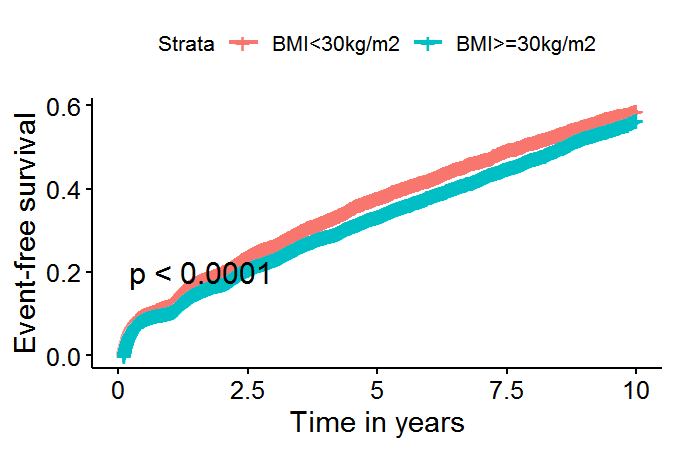

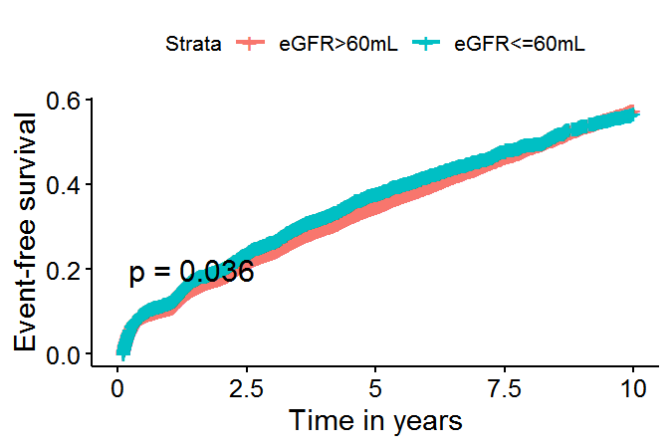


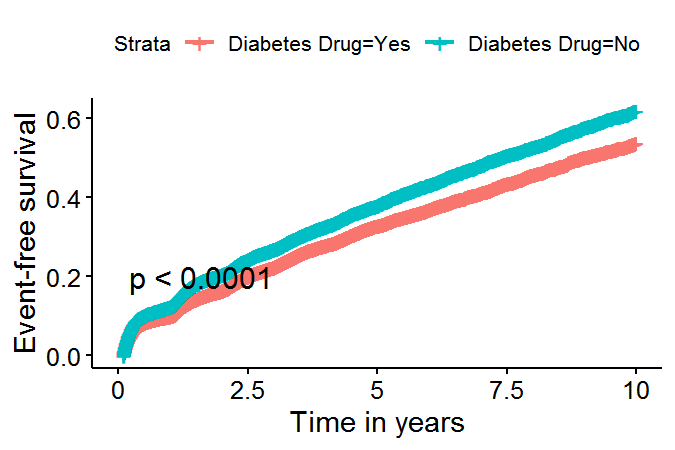

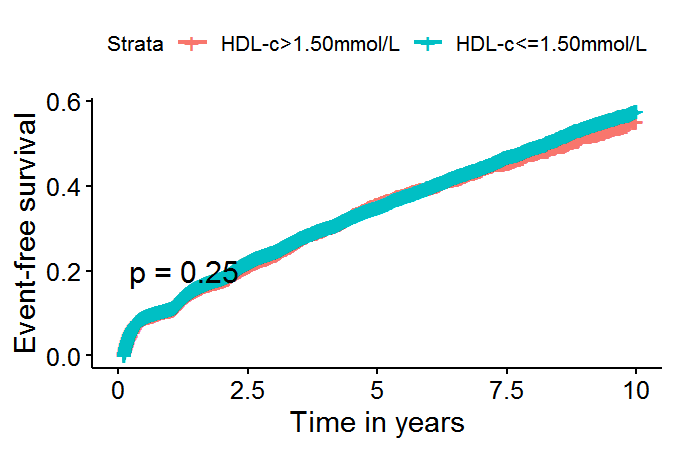


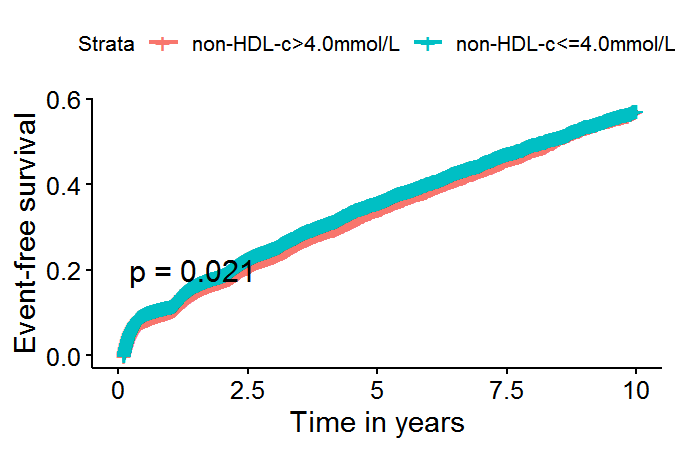


Figure 5. Cumulative Incidence Function (CIF) plots of NLR and covariates for 10-year DR incidence in Tayside and Fife (n=23,531)


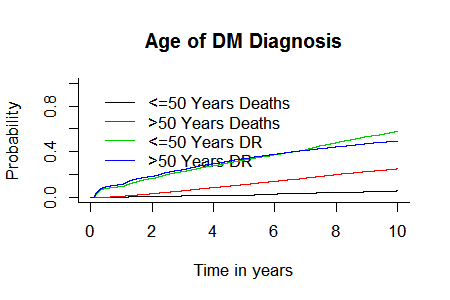

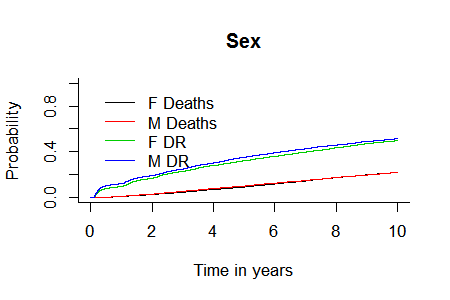


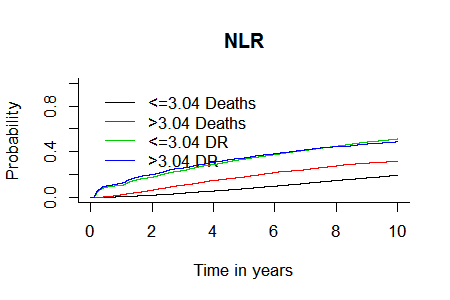

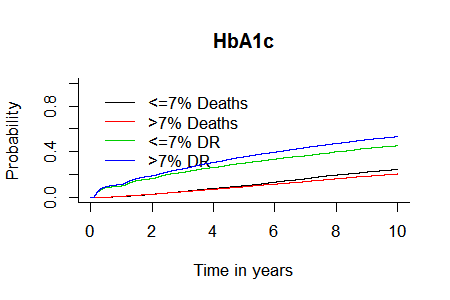


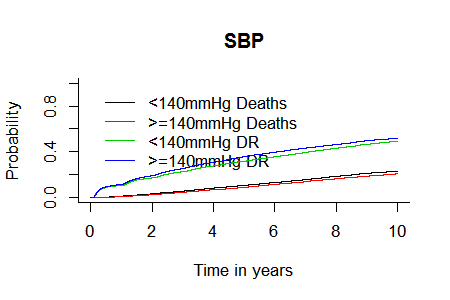

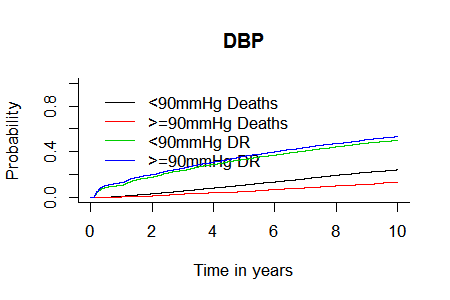


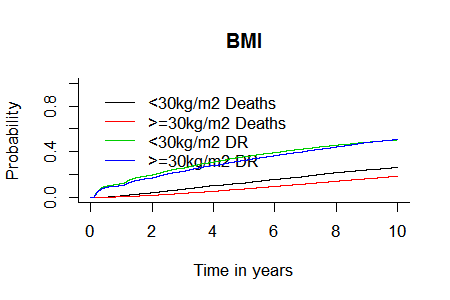

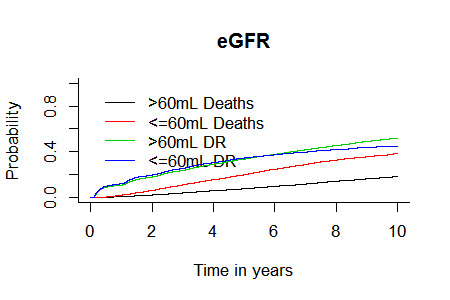


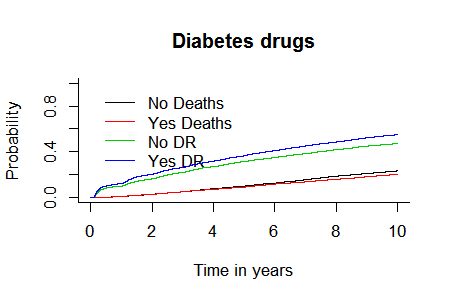

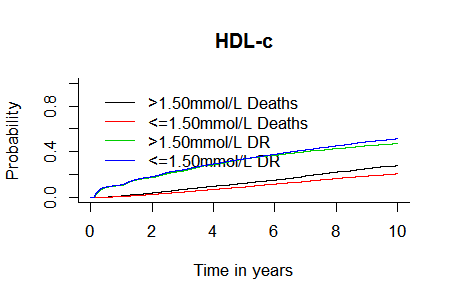


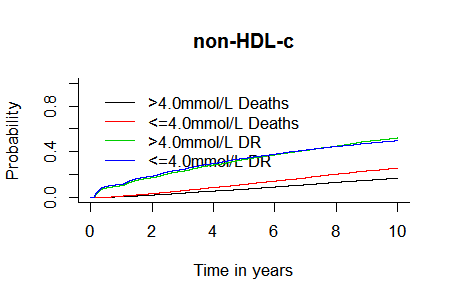

Supplement: Supplementary file 1 — Additional file 1: Figure S1. Study flow diagram. Figure S2. Overall Survival for the incidence of DR in Tayside and Fife. Figure S3. Survival for the incidence of DR in Tayside and Fife for the analysed cohort. Figure S4. Event-free survival (EFS) plots of NLR covariates for 10-year DR incidence in Tayside and Fife. Figure S5. Cumulative Incidence Function (CIF) Plot of NLR and covariates for 10-year DR incidence in Tayside and Fife. [file 12916_2023_2976_MOESM1_ESM.docx]
